# Characterization of Silver Nanoparticles under Environmentally Relevant Conditions Using Asymmetrical Flow Field-Flow Fractionation (AF4)

**DOI:** 10.1371/journal.pone.0143149

**Published:** 2015-11-17

**Authors:** Min-Hee Jang, Seungho Lee, Yu Sik Hwang

**Affiliations:** 1 Future Environmental Research Center, Korea Institute of Toxicology, Jinju, 660–844, Republic of Korea; 2 Department of Chemistry, Hannam University, Daejeon, 305–811, Republic of Korea; 3 Human and Environmental Toxicology Program, University of Science and Technology (UST), Daejeon, 305–350, Republic of Korea; Oregon State University, UNITED STATES

## Abstract

The development of methods to monitor manufactured nanomaterials in the environment is one of the crucial areas for the assessment of their risk. More specifically, particle size analysis is a key element, because many properties of nanomaterial are size dependent. The sizing of nanomaterials in real environments is challenging due to their heterogeneity and reactivity with other environmental components. In this study, the fractionation and characterization of a mixture of polyvinylpyrrolidone-coated silver nanoparticles (PVP-AgNPs) of three different sizes were investigated using asymmetrical flow field-flow fractionation (AF4) coupled with UV-Vis spectrophotometry. In particular, the effects of electrolyte composition and natural organic matter (NOM) on the particle size and stability were evaluated. The fractogram peaks (i.e., stability) of three different AgNPs decreased in the presence of both 10 mM NaCl and 10mM CaCl_2_, while increased with increasing concentration of humic acid (HA). In addition, the hydrodynamic diameters of AgNPs in both electrolytes slightly increased with an increase of HA concentration, suggesting the adsorption (coating) of HA onto the particle surface. It is also interesting to note that an increase in the particle size depended on the types of electrolyte, which could be explained by the conformational characteristics of the adsorbed HA layers. Consistent these results, AgNPs suspended in lake water containing relatively high concentration of organic carbon (TOC) showed higher particle stability and larger particle size (i.e., by approximately 4nm) than those in river water. In conclusion, the application of AF4 coupled with highly sensitive detectors could be a powerful method to characterize nanoparticles in natural waters.

## Introduction

Nanotechnology is a rapidly developing field with the significant applications of nanomaterial in various industrial productions [1]. The increased use of nanomaterials brings concerns for environmental risk with the potential release of nanomaterials into the environment. Characterization of manufactured nanomaterials is one of the important areas to understand their potential environmental risks [2]. Moreover, particle size analysis is a key element, because many properties of nanomaterial are size-dependent. However the measurement of particle size under environmentally relevant conditions is challenging due to the heterogeneity and broadness in size distributions of nanomaterials. In addition, water constituents (e.g., electrolyte composition) as well as natural organic matter (NOM) are likely to change the surface properties of nanomaterials and lead to aggregation and subsequent sedimentation. Therefore it is essential to evaluate the effect of water chemical composition on the particle size and stability.

Numerous methods for characterizing nanoparticles have been developed. Commonly used techniques for size characterization are dynamic light scattering (DLS) and electron microscopy such as scanning electron microscopy, transmission electron microscopy (TEM) and atomic force microscopy. However, the biased response of DLS for polydisperse samples toward larger particle size and concern about artifacts introduced by the sample preparation steps using electron microscopy limit the applicability of these methods in the environment, and calls for alternative techniques [3–5]. Recently, field-flow fractionation (FFF) which is a family of separation techniques has been shown to be a robust method for the analysis of nanomaterials in aqueous matrices [6–7]. Asymmetrical flow-field flow fractionation (AF4) is a member of FFF family that provides separation of particles based on the interaction between cross flow field forces and their translational diffusion [8]. Since particle retention time is a function of its diffusion coefficient, hydrodynamic diameter can be derived from Stokes-Einstein equation [9].

Baalousha et al. published a review article on applications of FFF coupled with different detection techniques [10]. In recent, Romer et al. described a method using AF4 to characterize AgNPs in exposure media for aquatic toxicity tests [11]. In this study, applicability of AF4 to characterize the behavior of AgNPs in different dilutions of the media was evaluated. Bolea et al. described AF4 with ICP-MS detection for the analysis of AgNPs in consumer products, indicating that the AF4 separation conditions affect the stability, recovery and resolution of the analyte [12]. Poda et al. also employed AF4-ICP/MS to study the particle size distribution of AgNPs extracted from biological receptors after exposure [5]. This study described AF4-ICP/MS is applicable to environmentally relevant particle concentrations (μg/L). Cumberland et al. utilized an AF4 method to characterize the particle size distribution of AgNPs in aqueous systems that were varied in terms of NOM, pH and ionic strength [4]. Collectively, these articles show the utility of AF4 with diverse detectors, but there are no published data on the analysis of heterogeneous nanoparticle sample in real environmental samples having different water composition. In addition, details of the effects of electrolytes and NOM on the particle size and stability remain poorly understood.

In this study, we evaluate the fractionation and characterization of polyvinylpyrrolidone-coated silver nanoparticles (PVP-AgNPs) mixture of three different sizes using AF4 coupled with UV-Vis spectrophotometry and DLS. The objectives of this study were 1) to confirm the capability of AF4 to separate heterogeneous nanoparticles and 2) to evaluate the effect of electrolyte composition and NOM on the particle properties (i.e., particle size and stability) and, finally 3) to investigate the applicability of AF4 to analyze the nanoparticles in natural environmental waters.

## Materials & Methods

In this study, we dispersed the Ag nanoparticles in samples taken from different media associated with river and lake. Although the water sample were collected, all studies were carried out in laboratory (actually no field study). Therefore, no specific permissions were required for this study.

### Materials

Polyvinylpyrrolidone (PVP)-coated silver nanoparticles (AgNPs) with an average particle diameter of 30, 60, and 100 nm (manufacturer-provided particle size determined by TEM, size distribution (CV) < 15%) were purchased from Nanocomposix (Nanoxact, San Diego, CA). The particles were generally mono-dispersed in size and the concentration of each suspension was 0.02 mg/mL as provided by the manufacturer. All AgNPs stock suspensions were stored in light protected PE vessels at 4°C. Sodium chloride (NaCl, ≥ 99.0%) and calcium chloride dehydrate (CaCl_2_, > 99.5%) were purchased from Sigma (St. Louis, USA) and Sigma Aldrich (Tokyo, Japan), respectively. Sodium azide (NaN_3_ > 99.5%) was obtained from Fulka (Bangalore, India). Aldrich humic acid (HA) (Munich, Germany) was used as the surrogate for natural organic matter (NOM). To obtain the HA stock solution, 50 mg HA powder was dissolved into 500 mL high purity Milli-Q water and pH was adjusted to 7.0 using 0.1 M sodium hydroxide and 0.1 M chloric acid. The HA stock solution was stirred overnight and filtered through a 0.45 μm cellulose acetate membrane filter to remove undissolved HA. The concentration of the stock solution was 31.6 mg/L total organic carbon (TOC), determined by a Schimadzu TOC analyzer (TOC-VCPN, Schimadzu, Japan) which utilized a high temperature oxidation procedure with non-dispersive infrared detection. Natural water samples were collected at two different sites including river water (Nam-river, Jinju, Korea) and lake water (Yugae-gi, Jinju, Korea). The water samples were filtered through 0.1 μm membrane filters to remove natural nanoparticles into negligible levels and stored in a refrigerator until use.

### Sample Preparation

To demonstrate the separation and detection potential of the AF4 method (size measurement technique), a mixture of AgNPs of three different sizes was used in all study. For characterizing the behavior of AgNPs suspensions at different electrolyte, NaCl and CaCl_2_ were chosen as they are the most abundant monovalent and divalent cations in natural freshwaters. HA was used to investigate the effect of NOM on the behavior of AgNPs suspension. The tested suspensions with the desired solution (i.e., cations and NOM) were prepared by mixing appropriate volumes of the NaCl or CaCl_2_ stock solutions, HA stock solutions, and AgNPs mixture suspensions. The final concentration of each particle for AF4 measurements was 3.3 mg/L to give satisfactory separation and detection. AgNPs mixtures suspended in natural water were also prepared by diluting the AgNPs mixture with the river or lake water. All prepared suspensions were equilibrated for 24 h at room temperature, and the samples were weakly sonicated before the analysis. All of the samples had a pH within 6.5 ± 0.2.

### AF4

The AF4 system used was an AF2000 MT model purchased from Postnova Analytics Inc. (Germany). Sample detection was achieved with an online UV-Vis detector (SPD-20A, Shimadzu, Japan) and DLS detector (Nano-ZS90, Malvern, UK). The UV-Vis detector was operated at the wavelength of 410 nm to detect all of the AgNPs, although the shift in wavelength maxima with increasing AgNP size was observed (see S1 Fig). The channel was equipped with a 10 kDa regenerated cellulose membrane and 350 μm channel spacer. For all AF4 analysis, the carrier liquid was high purity Milli-Q water containing 0.02% sodium azide. Samples were injected via a manual injector valve (Rheodyne switching valve, Japan). Channel flow of 2.0 ml/min and cross flow of 1.0 ml/min was set to obtain good separation. Additional details of the AF4 separation conditions are listed in S1 Table. The channel volume was calculated applying FFF theory as previously described by Baaloush et al. [13] using 21 and 43 nm polystyrene latex (Postnova Analytics Inc., Germany), which were detected by the UV detector at 254 nm. Diffusion coefficients were calculated based on the standard FFF theory and converted to size using the Stokes-Einstein relationship [14]. Further details regarding FFF theory are given in S1 File. For off-line collection of AF4 fractions, a fraction collector (Retriever 500, Tledyne Isco, USA) was connected with the AF4 system. The collected fractions from AF4 system were analyzed by TEM to confirm the particle size.

### TEM

TEM images for mixed and separated AgNPs suspension were obtained to confirm the individual particle size. Before the analysis, the mixed AgNPs suspensions were diluted approximately 10 times with HPLC grade methanol (Burdick&Jackson, Korea) to achieve optimal surface coverage. The AgNPs suspensions separated from AF4 were concentrated approximately 20 times as AF4 technique dilutes the samples on the channel. About 10 μL of sample was dropped on the carbon coated copper TEM grid (CF200-Cu, Electron Microscopy Science, USA) and allowed to dry for the analysis. Images were obtained from an electron microscope (JEM-2010, Jeol Ltd., Japan) and recorded using Gatan software (Gatan Microscopy Suite^®^ 2.0, Gatan, Japan).

### DLS and zeta potential

Particle size measurements of AgNPs mixture were also conducted with a zetasizer (Nano-ZS90, Malvern, UK) in disposable cuvettes and average hydrodynamic diameter was determined by taking an arithmetic average of 10 runs. Particle sizes of AgNPs separated by AF4 were measured in flow mode of DLS detector using a quartz flow-through cell (Hellma, Germany). For these experiments, AF4 system was directly interfaced to a zetasizer without channel split and the detector flow was set to 1.0 ml/min for all fractions. The surface charge of AgNPs was determined by zeta potential measurements with the same equipment. All experiments were carried out in triplicate and the results presented are the average measurements of the runs with standard deviation.

## Results and Discussion

### Comparison of Size Measurement Techniques (TEM, DLS, AF4)

**Fig 1:**
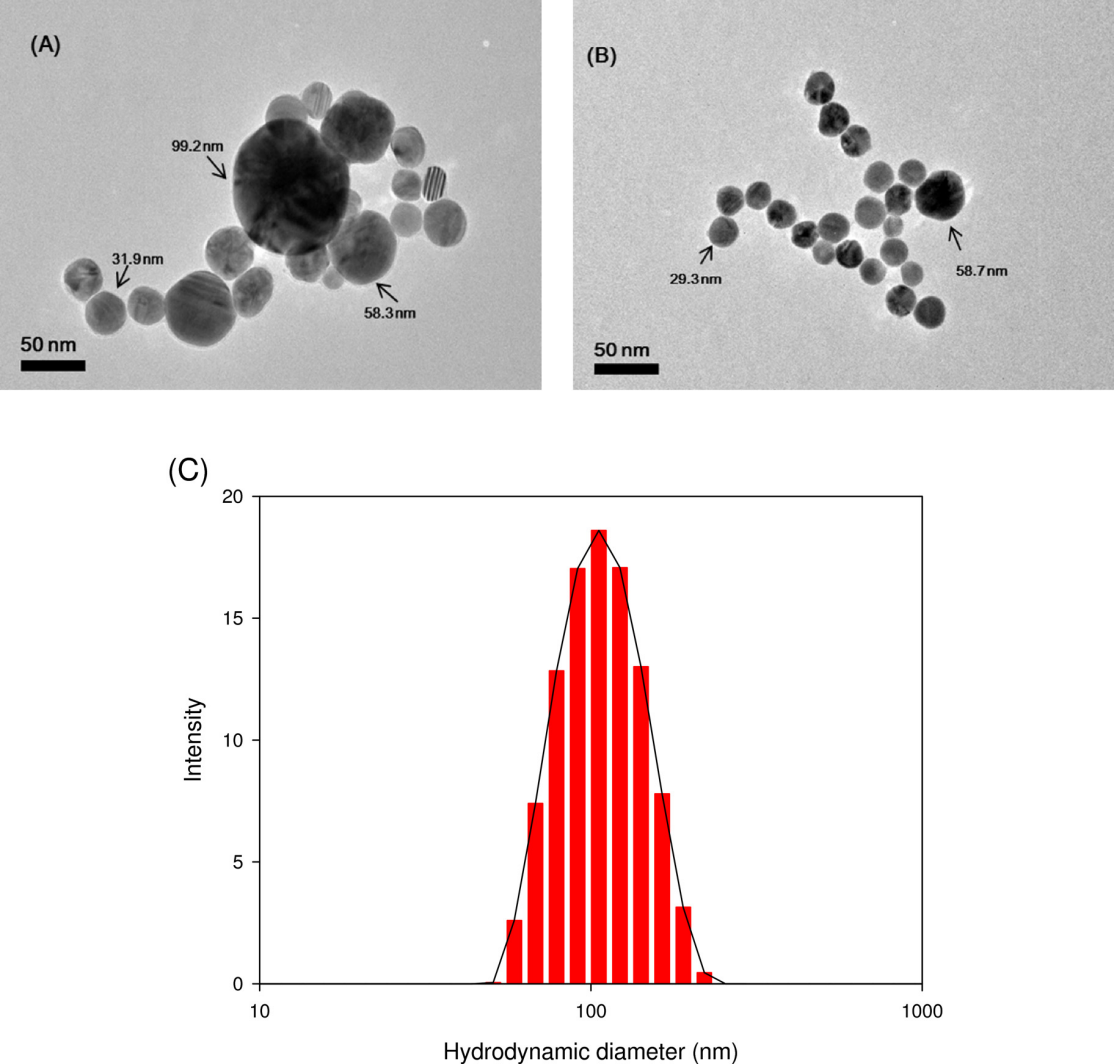
TEM image (A and B) and particle size distribution determined by batch-DLS (C) for AgNPs mixture.

The TEM image shown in Fig 1 (A) and 1 (B) demonstrates the spherical shape and heterogeneous particle size distribution of AgNPs mixture. The particle size determined by TEM image was close to the size reported by the manufacturer. It is known that the probability to observe the larger particles is thousands of times lower than smaller one (particle number is proportional r^3^) [15]. Consistent with this, AgNPs with the particle size of 100 nm were not easily observed in most of TEM images (e.g., Fig 1(B)). To analyze the overall AgNPs mixture (Fig 1(A)), much effort was needed and this led to a time-consuming procedure. Even though TEM images are fairly representative for the particles in suspension, counting as many particles as possible is needed to obtain a representative size distribution, especially in case of unknown samples with highly polydispersed particles. Particle size distribution determined by batch mode of DLS for AgNPs mixture was shown in Fig 1(**C**). Although the sample was a mixture of AgNPs of three different sizes, DLS intensity analysis gave one broad peak and was weighted toward the larger particles (z-average size of 99.1 nm). DLS measurement is known to be more sensitive to larger particles [3]. These results suggest that DLS measurement may not be accurate for polydisperse samples due to its nature to respond toward larger particles.

**Fig 2:**
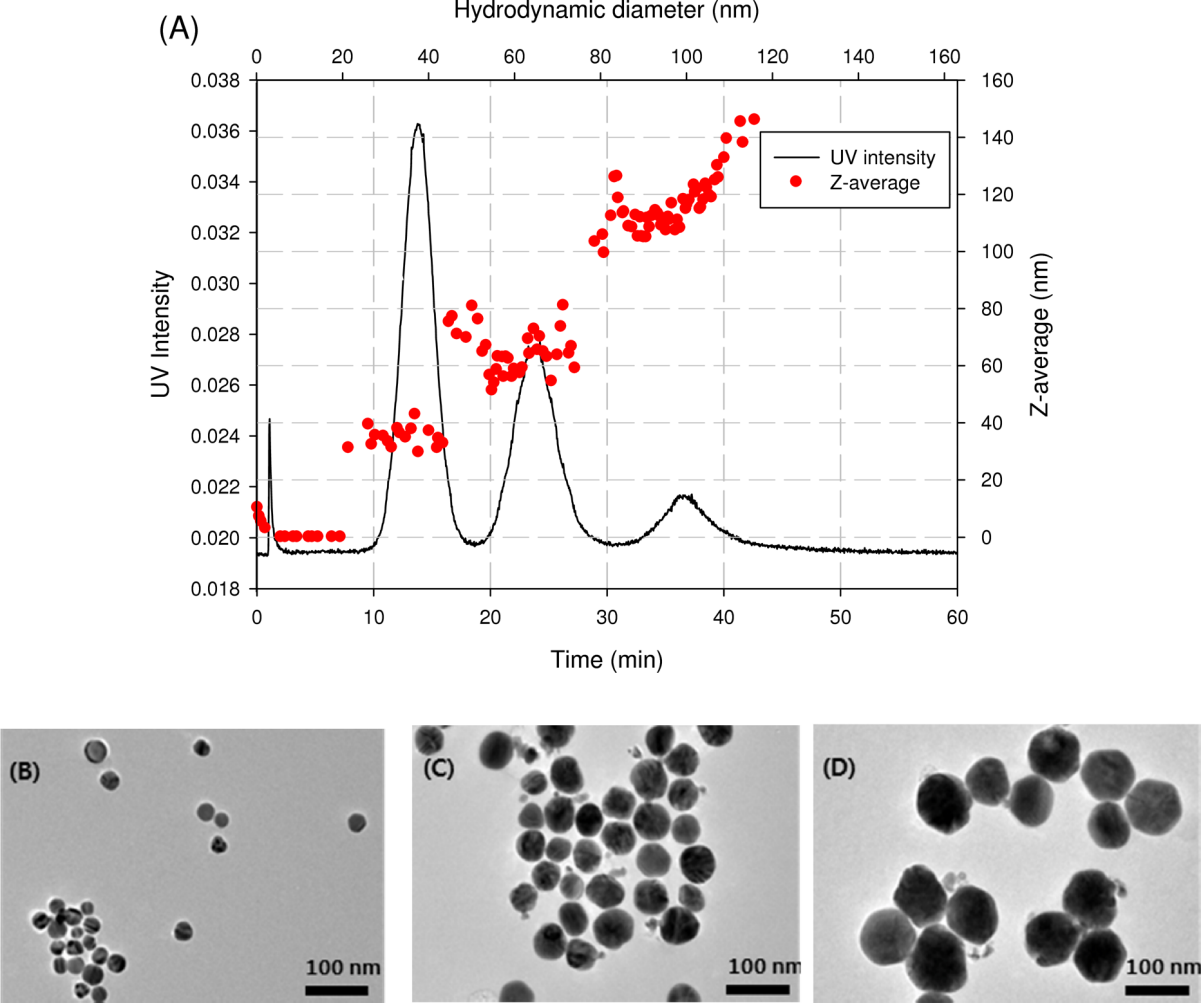
AF4 fractogram of a AgNPs mixture (A) and TEM images of fractions collected at elution time of 13 (B), 23 (C), and 36 min (D), respectively.

An AF4 fractogram of AgNPs mixture obtained under the standardized processing conditions (S1 Table) was shown in Fig 2(A). The AF4 system produced clearly defined three peaks with high resolution and appropriate recovery (107.1 ± 17.6%), which is deemed to excellent recovery for traditional analysis. Hydrodynamic diameters of AgNPs derived from UV/Vis retention time using FFF equation and Stokes-Einstein equation (S1 File) were 37.3, 63.9, and 99.1 nm, respectively. Particle sizes measured by online-DLS were 34.8 ± 3.4, 64.7 ± 7.5, and 114.4 ± 7.3 nm, respectively. The particle sizes determined by both UV-Vis and DLS detector were slightly larger than the nominal sizes, probably because both methods measure a hydrodynamic size, rather than a physical size. The fractions collected from AF4 system were further analyzed by TEM. The TEM images of the fractions collected at the elution time of 13, 23, and 36 min (maximum absorbance) were presented in Fig 2B, 2C and 2D. The particle sizes measured from TEM images were 28.8 ± 6.4 (n = 42 particles), 58.0 ± 8.1 (n = 66 particles), and 96.6 ± 9.6 nm (n = 16 particles), respectively, which are in good agreements with the values reported from the manufacturer.

### Effect of solution composition on AgNP size

**Fig 3:**
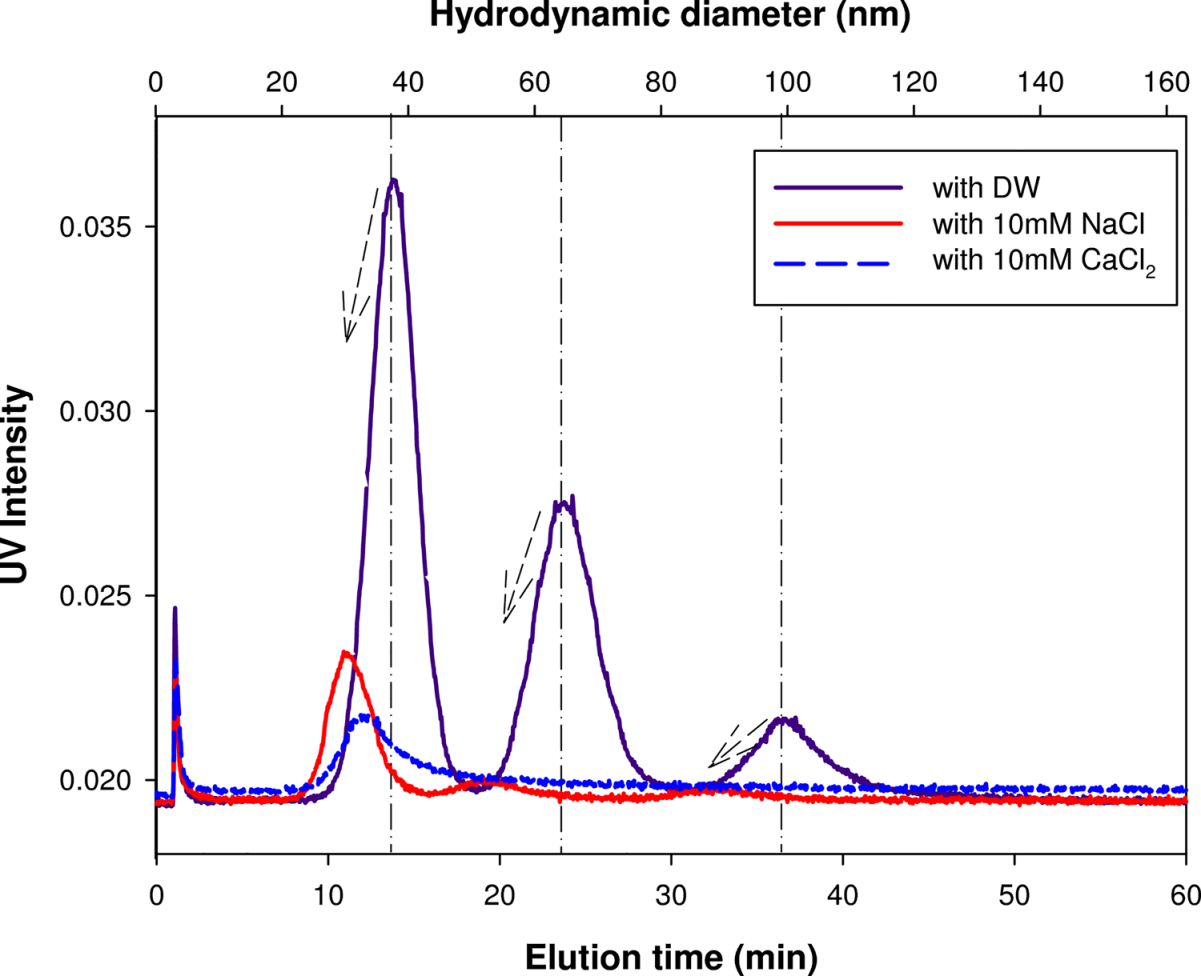
AF4 fractograms of AgNPs mixture suspended in various types of electrolytes.

The AgNPs mixture suspended in aqueous solution of 10 mM NaCl or 10mM CaCl_2_ with the presence/absence of model natural organic matter (NOM) was characterized by AF4 in order to evaluate the effects of solution composition on the AgNPs size and stability. Fig 3 shows the AF4 fractograms of the AgNPs mixture in the presence of different electrolytes (i.e., 10 mM NaCl and 10mM CaCl_2_). For easy of comparison, the AF4 fractogram of the same AgNPs mixture suspended in DW (Fig 2) was also presented. In the presence of 10 mM electrolytes, UV intensities were significantly decreased, which indicates formation and sedimentation of large aggregates during analysis. Clearly, the decrease in UV intensity was more prominent in 10mM CaCl_2_ than in 10 mM NaCl. As the ionic strength increases, the electrical double layer around the AgNPs might be more tightly compressed by screening the surface charge of the AgNPs (i.e., -33.6 ± 2.7 mV in DW, -23.5 ± 3.6 mV in 10 mM NaCl and -8.7 ± 0.9 mM in 10 mM CaCl_2_), which allows a greater degree of particle-particle interaction resulting in an increase in the level of aggregation and potential for particle settling [16].

It is noted that the hydrodynamic sizes of particles measured in the presence of electrolyte were slightly reduced in comparison with those in DW. This is supposed to be due to the compression of the electrical double layer in the presence of weak cations. According to the Poission-Boltzmann equation, the electric double layer thickness (the Debye length (k^-1^)) can be affected by ionic concentration in medium. A similar result has been reported by Cumberland and Lead [14], showing a reduction in AgNPs size from 25–30 nm to below 20 nm in the presence of cations. In our study, AF4 may not show all of particles during the analysis of aggregates [17], but it seems that AF4 can detect a fine alteration of particles which are still small enough to stay dispersed in suspension.

**Fig 4:**
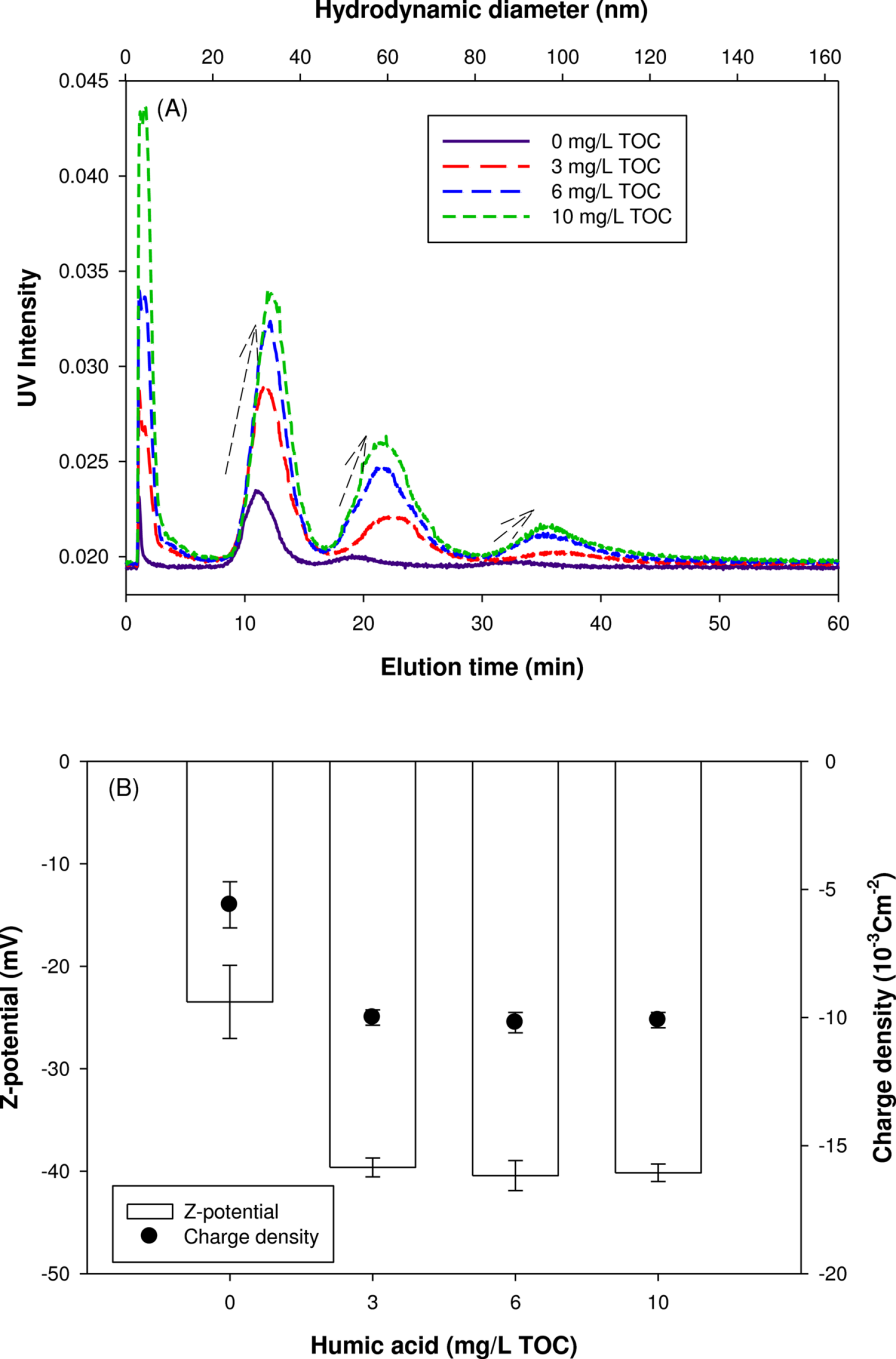
NOM effect; AF4 fractograms (A) and zeta potential (B) of AgNPs mixture suspended in 10 mM NaCl.

The presence of NOM changed the AF4 elution of nanoparticles. In 10 mM NaCl, the UV signal intensity gradually increased with the increase of HA concentration, indicating the increased particle stability (Fig 4(A)). The presence of NOM is known to bring a surface coating of nanoparticles with NOM molecules [18, 19]. The adsorption of organic macromolecules onto nanoparticles could inhibit the aggregation potential of nanoparticles and thus enhance their stability due to electrostatic and steric stabilization [20]. Shift in the peak maximum toward larger sizes was also observed in the presence of HA, indicating the likely formation of a surface coating with an average thickness of 2.6 ± 0.5 nm (1.9, 2.7, and 3.2 nm for 30 nm AgNPs with 3, 6, and 10 mg/L of HA, respectively). According to previous studies, the sizes of HA measured by fluorescence correlation spectroscopy [21] and atomic force microscopy [22] were from 0.5 to 2.5 nm, which is in a good agreement with our result. Fig 4(B) presents the effect of HA concentration on the surface charge of AgNPs in 10mM NaCl. In the presence of HA, the absolute value of zeta potential was significantly increased from 23.5 ± 3.6 to 40.2 ± 0.9 mV. However, above 3 mg/L HA, the change in zeta potential was not significant. This result suggests that the stabilization of AgNPs with NOM resulted from not only electrostatic effect but also steric hindrance effect.

**Fig 5:**
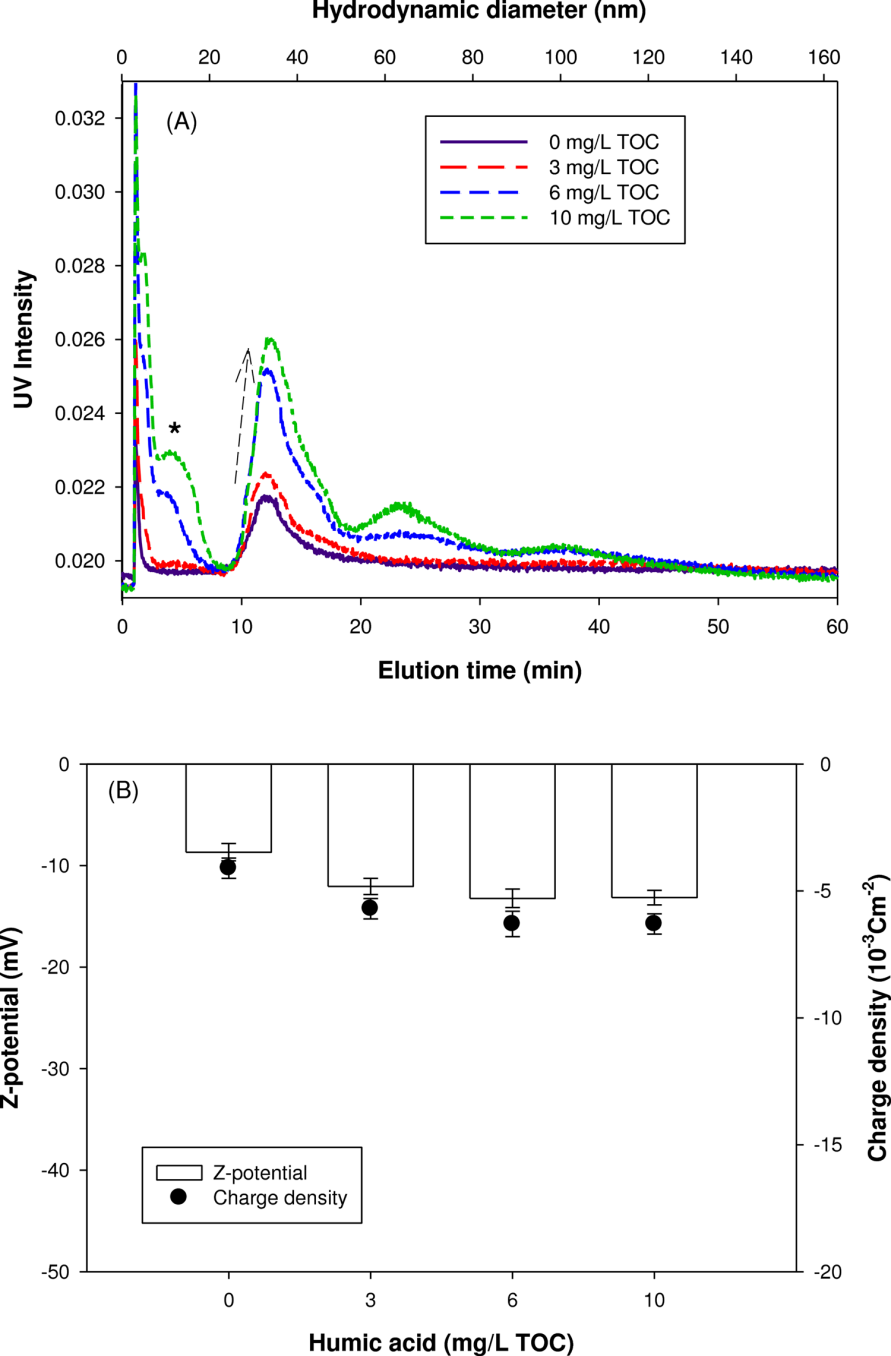
NOM effect; AF4 fractograms (A) and zeta potential (B) of AgNPs mixture suspended in 10 mM CaCl_2_.

AF4 fractograms of AgNPs suspended in 10 mM CaCl_2_ are presented in Fig 5(A) as a function of HA concentration. Similar to the trend observed in Fig 4(A), the UV intensity gradually increased with increasing HA concentration, indicating that HA also enhanced the stability of AgNPs suspended in 10 mM CaCl_2_ solution. In comparison with the results in 10 mM NaCl, a smaller shift in the peak maximum toward larger sizes (0.3, 0.7, and 1.0 nm for 30 nm AgNPs with the 3, 6, and 10 mg/L of HA, respectively) and a peak broadening (due to a shoulder shown near 45nm) were observed. This behavior could be explained by the conformational characteristics of adsorbed NOM layer in the presence of Ca^2+^. In the presence of multivalent cations (e.g., Ca^2+^), the intra-molecular contraction or intermolecular aggregation of NOM could occur due to the charge neutralization and/or cation bridging [23, 24]. In our study, this hypothesis was also supported by analyzing the surface charge density change resulted from HA adsorption. Comparing with the AgNPs suspended in 10 mM NaCl (Fig 4(B)), a smaller change in surface charge in 10 mM CaCl_2_ was observed with HA concentration (Fig 5(B)), indicating that the electrostatic interaction between Ca^2+^ and negative functional groups in HA and the complexation/cation bridging effect of Ca^2+^ with carboxylic groups in HA would impede the increase in the surface charge. It seems that the peak broadening and a shoulder to the void peak found at approximately 5nm likely resulted from the formation of cation bridging and the aggregation of unadsorbed HA molecules, respectively. The observed changes in AF4 fractrograms require a more comprehensive investigation. Since the variation of the zeta potential with the NOM concentration was small, the increased stability could be mainly attributed to the steric hindrance of the NOM layer.

### Application to natural environmental water

**Fig 6:**
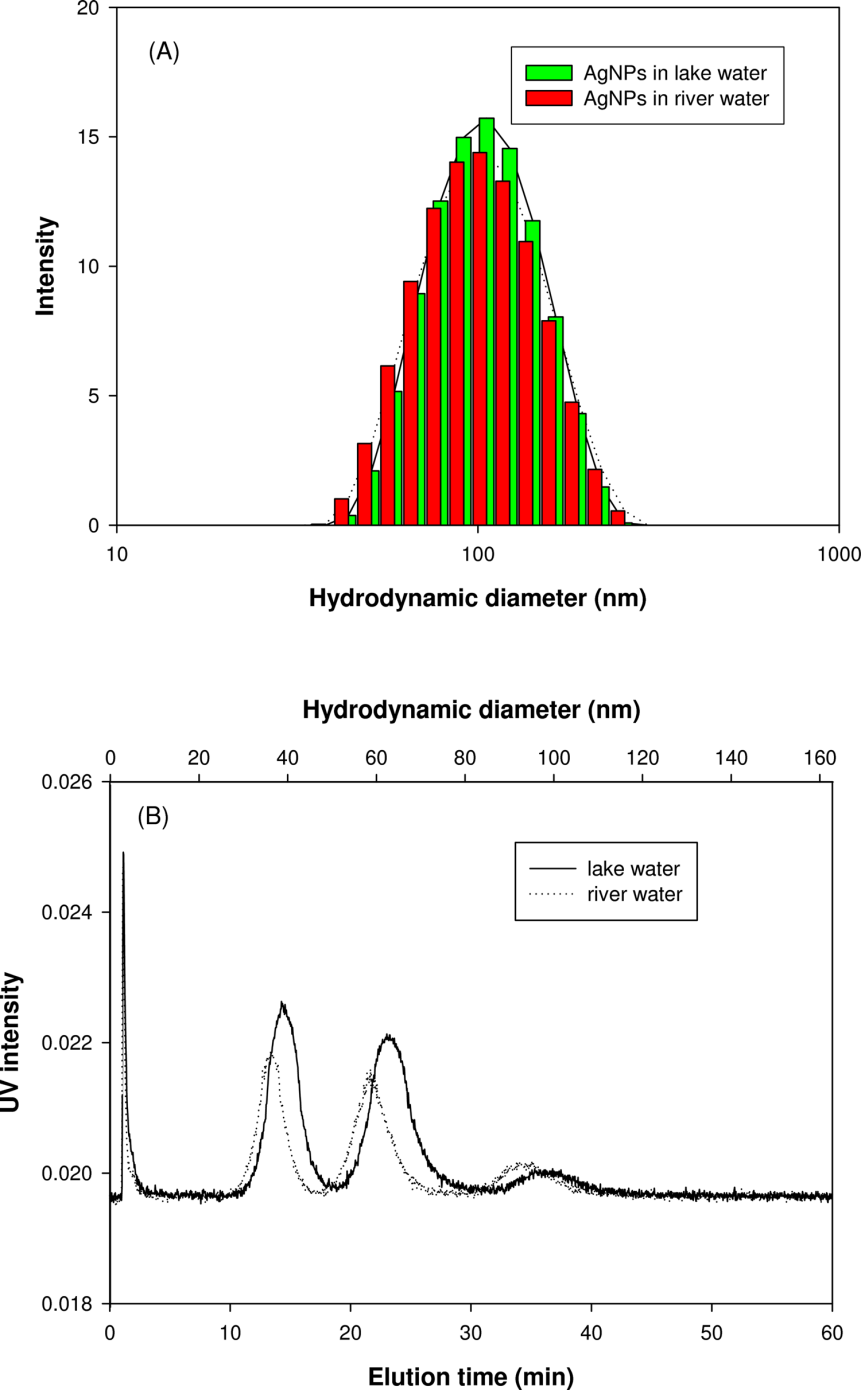
Particle size distribution determined by batch-DLS (A) and AF4 fractograms (B) of AgNPs mixture suspended in natural water.

The applicability of AF4 to analyze the nanoparticles in natural environmental water was determined by spiking the AgNPs mixture of three different sizes into natural environmental water samples. Fig 6 shows the size distributions obtained by batch mode of DLS (Fig 6(A)) and AF4 fractograms of AgNPs mixture spiked into two different environmental water (Fig 6(B)), which were a lake water (5.4 mg/L TOC, 180 μS/cm conductivity, pH 7.6) and river water (3.0 mg/L TOC, 145 μS/cm conductivity, pH 7.7). Although batch-DLS analysis yielded one broad size distribution, AgNPs of three different sizes were well separated by AF4. Interestingly, the AF4 fractogram of the AgNPs mixture dispersed in lake water had higher UV intensities than those in river water. This result is linked to the effect of NOM on the properties of nanoparticles (electrostatic and steric effect). Even though the electric conductivity (as an indicator of ionic strength) of lake water was higher than the river water, it is likely that the particles remain more stable in lake water due to the NOM effect. Peak shift toward larger sizes (e.g., 36nm in river water vs. 39nm in lake water) was also observed indicating the formation of surface coating with NOM. In conclusion, AF4 analysis showed high potential for characterization of nanoparticles suspended in natural water. Further studies are needed to investigate the effect of various water chemistry on the properties of nanoparticles, since there are various types and sources of NOM (i.e., Fulvic and Humic acid) and electrolytes in natural water.

## Supporting Information

S1 Fig UV-vis absorption spectra of 30, 60, and 100 nm silver nanoparticles in water. (TIF)

S2 Fig Fractograms of humic acid in 10 mM CaCl_2_. (TIF)

S1 File FFF and Stokes-Einstein equation. (PDF)

S1 Table Analytical parameters used for separation of AgNPs by AF4 system. (PDF)
