# Supplementary figures and images for: Characterization of Silver Nanoparticles under Environmentally Relevant Conditions Using Asymmetrical Flow Field-Flow Fractionation (AF4)
Source: PLoS One. 2015 Nov 17;10(11):e0143149. doi: 10.1371/journal.pone.0143149 (PMC4648590; doi:10.1371/journal.pone.0143149)

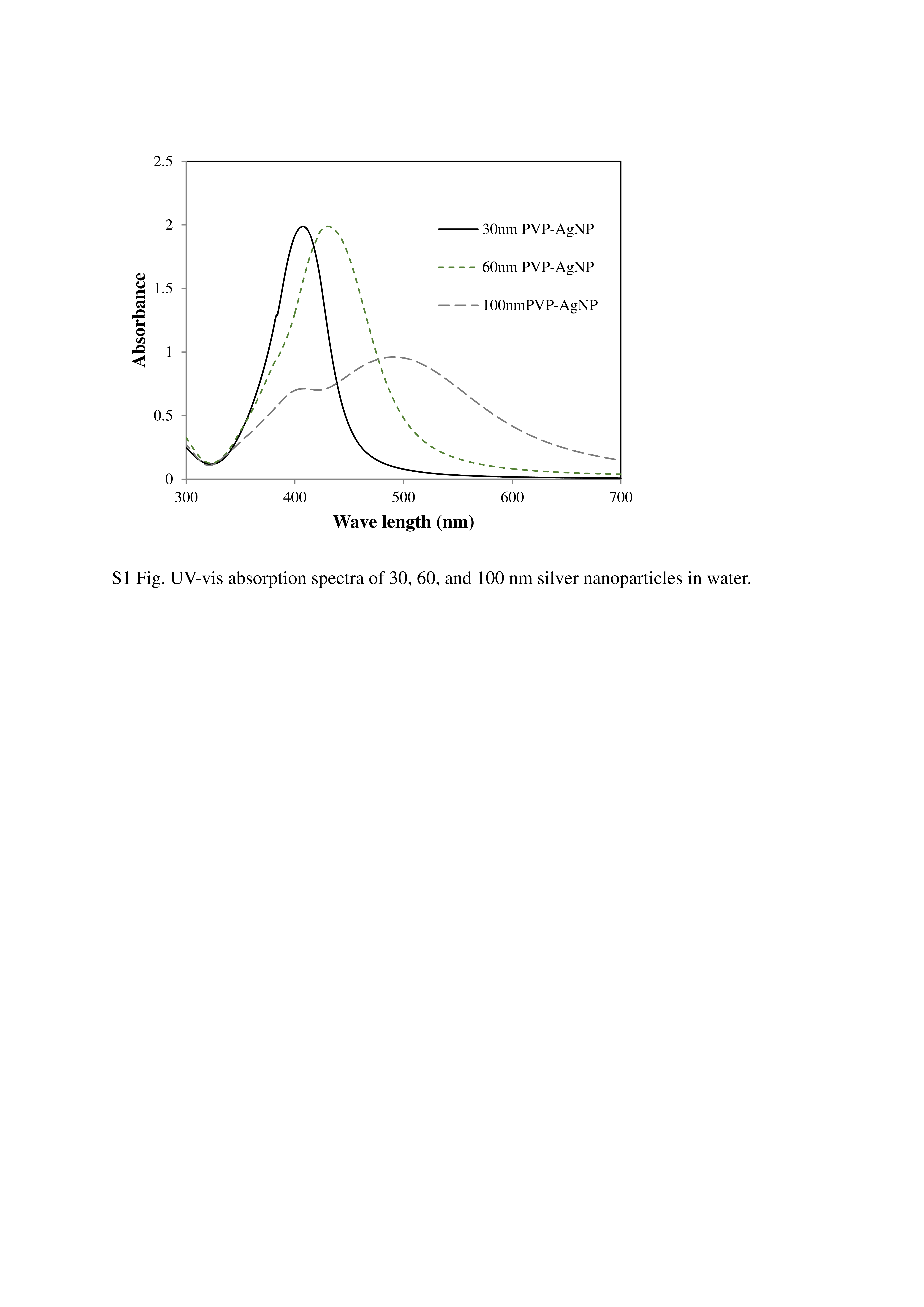

Supplement: S1 Fig — (TIF) [file pone.0143149.s001.tif]

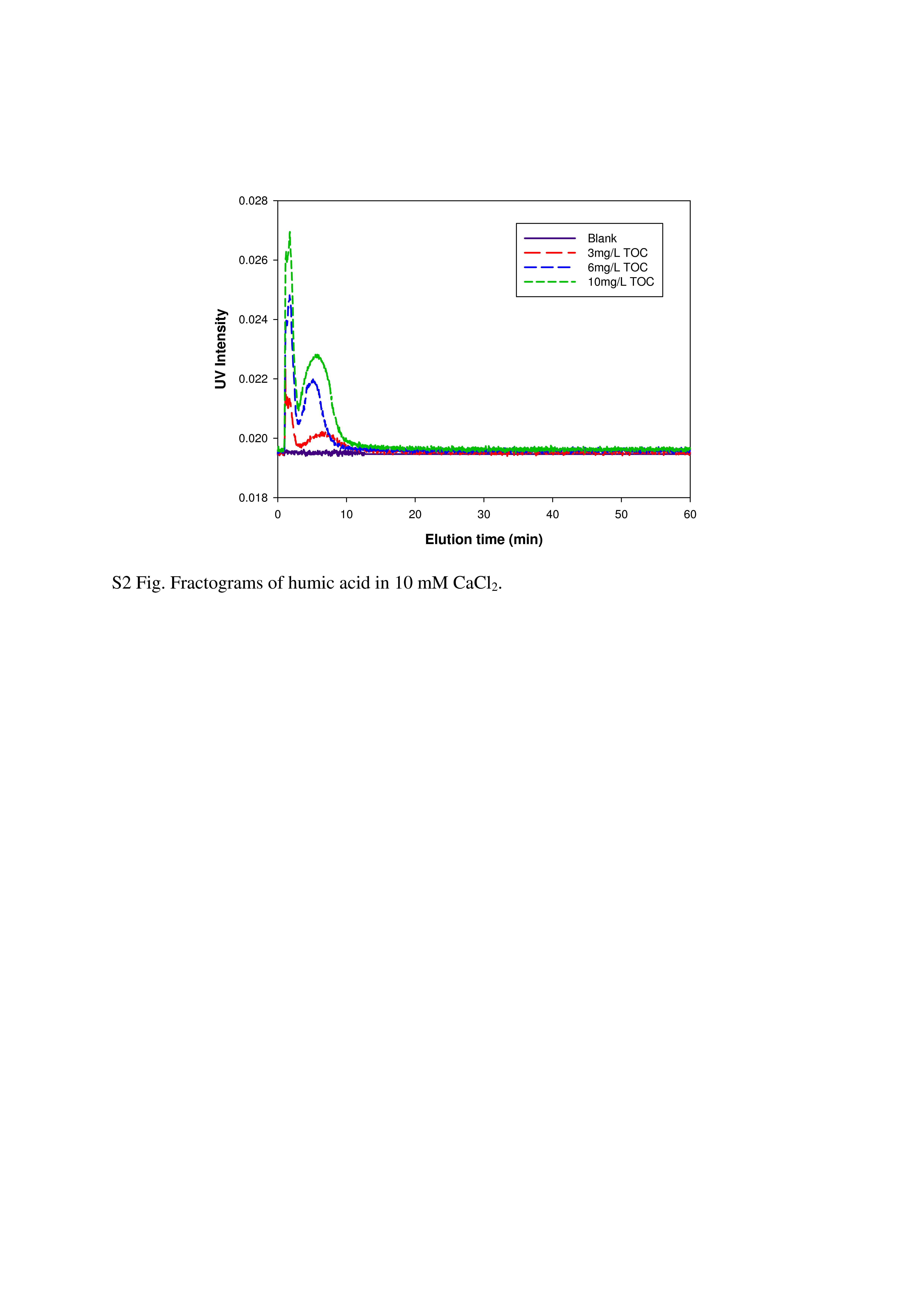

Supplement: S2 Fig — (TIF) [file pone.0143149.s002.tif]
