# Supplementary material for: Characterization of Silver Nanoparticles under Environmentally Relevant Conditions Using Asymmetrical Flow Field-Flow Fractionation (AF4)
Source: PLoS One. 2015 Nov 17;10(11):e0143149. doi: 10.1371/journal.pone.0143149 (PMC4648590; doi:10.1371/journal.pone.0143149)
Supplement: S1 File — (PDF) [file pone.0143149.s003.pdf]

S1 File. FFF and Stokes-Einstein equation.

One of the advantages of FFF is that elution time under identical conditions is related to particle size and follows a linear correlation. To calculate the modal size of nanoparticles, a simplified form of the FFF equation (Eq. (1)) and Stokes-Einstein equation (Eq. (2)) were used.

$$t_r = \frac{w^2}{6Dh\,(1+F_c/F_{out})} \qquad (1)$$

$$\mathrm{D} = \frac{K_B T}{6\pi\eta r} \qquad (2)$$

Where, t_r, W, D, F_c, F_{out}, K_b, T, η, and r are retention time, channel height (thickness of spacer), diffusion coefficient of the particles, cross flow, channel flow, Boltzman's constant, temperature (K), dynamic viscosity of fluid (e.g., 0.01 gm cm^{-1} s^{-1} for water) and radius of particle, respectively.
