# Supplementary material for: Characterization of Silver Nanoparticles under Environmentally Relevant Conditions Using Asymmetrical Flow Field-Flow Fractionation (AF4)
Source: PLoS One. 2015 Nov 17;10(11):e0143149. doi: 10.1371/journal.pone.0143149 (PMC4648590; doi:10.1371/journal.pone.0143149)
Supplement: S1 Table — (PDF) [file pone.0143149.s004.pdf]

S1 Table. Analytical parameters used for separation of AgNPs by AF4 system.

Detector flow rate:	1.0 ml/min	
Focus Step:	Injection flow	0.1 ml/min
	Cross flow	1.0 ml/min
	Injection time	8 min without transition time
	Injection vloume	200uL
Elution step:	Cross flow	1.0 ml/min with constant mode
	Elution time	80 min
